# A knowledge-driven framework for surgical safety check integration using speech recognition and speaker verification

**DOI:** 10.3389/fnins.2025.1726720

**Published:** 2026-01-12

**Authors:** Wen Shi, Rui Fan, Jian Hu, Jingrong Wang, Mei Bian, Wei Jiang

**Affiliations:** 1 Air Force Medical University Tangdu Hospital, Xi’an, China; 2 Department of Gastroenterology, The First Affiliated Hospital of Xi'an Jiaotong University, Xi’an, China

**Keywords:** surgical safety checklist, automatic speech recognition, speaker verification, knowledge-driven reasoning, operating room noise robustness

## Abstract

**Background:**

The WHO Surgical Safety Checklist reduces preventable errors, but real operating rooms are characterized by overlapping speech, high ambient noise, similar vocalizations, uncertain speaker identity, and occasional omission of checklist items. ASR-only systems that ignore identity constraints and semantic–temporal structure are therefore prone to misrecognition and incorrect verification.

**Method:**

We design an integrated verification framework that couples automatic speech recognition and speaker verification (ASR + SV) with a knowledge-driven rule engine derived from the WHO checklist. Multichannel audio is processed by a Conformer-based ASR module and an ECAPA-TDNN speaker verification model, after which a rule layer enforces consistency across the semantic content, speaker role, and checklist phase using an explicit ontology and conflict-resolution rules. The system generates real-time prompts in four states (“pass,” “fault,” “alarm,” “uncertain”). Performance is evaluated primarily in high-fidelity simulated operating-room scenarios with controlled noise levels, speaking distances, and multi-speaker interactions, using word error rate (WER), equal error rate (EER), checklist verification accuracy, and alarm rate. Three configurations are compared on the same held-out sessions—“ASR-only,” “ASR + SV,” and the full knowledge-driven method—and ablation experiments isolate the contribution of the rule layer.

**Results:**

Under medium-to-high noise and multi-speaker interference, the full framework reduced WER from 18.7% to approximately 13.5% and achieved an EER of about 3.1% relative to the ASR-only baseline. Checklist verification accuracy reached 93.8%, while the alarm rate decreased to roughly 2.7%. The knowledge layer corrected errors arising from homophones, accent drift, and role confusion by constraining “role–semantics–process” relations, and maintained robust performance at speaking distances up to 1.5 m and background noise of 60 dB. Residual failures were mainly associated with extreme speech overlap and unseen vocabulary, suggesting that lexicon adaptation and speech separation will be necessary for further gains.

**Conclusion:**

The proposed knowledge-driven ASR + SV framework jointly addresses semantic correctness and speaker identity while remaining interpretable, auditable, and suitable for embedded deployment. It provides a technical foundation for “time-out” and “operation review” functions in intelligent operating rooms. Because the present validation is based largely on simulated scenarios with limited real-world testing and no formal user or ethical evaluation, future work will focus on clinical pilot studies, integration with electronic medical records and multimodal OR data, and a deeper analysis of privacy, accountability, and workflow acceptance.

## 1 Introduction

Studies consistently show that the WHO Surgical Safety Checklist (WHO 19-item checklist) reduces perioperative complications and mortality, yet its implementation in the operating room (OR) remains fragile. Practical barriers include incomplete team participation, competing tasks, noisy environments, and variation in how protocols are applied. In a large multicenter trial, adoption of the checklist reduced in-hospital complication rates from 11.0 to 7.0% and mortality from 1.5 to 0.8% (Nakawala et al., 2017). With an estimated 234 million surgical procedures performed worldwide each year, even modest gains in checklist compliance translate into substantial population-level benefit. Nonetheless, recent sentinel event reports still attribute wrong-site and other preventable adverse events to issues such as improper checklist placement, incomplete or rushed time-out procedures, distracted attention, and lack of team consensus (Meng et al., 2025). The Joint Commission’s 2023 review noted that “improper surgical procedures” remained among the top three serious events, with a 26% increase over 2022, frequently citing inadequate time-outs, distraction, and unresolved disagreement as root causes. U. S. data suggest that wrong-site or wrong-procedure events occur between 0.09 and 4.5 per 10,000 cases annually (Pandithawatta et al., 2024).

From an environmental standpoint, the OR is a high-noise, multi-speaker setting. Systematic reviews report that equivalent continuous noise levels rarely fall below 50 dB and more commonly range between 51 and 79 dB, with most procedures taking place around 53–63 dB (Zhang et al., 2025). In specialties such as orthopedics, the use of saws, drills, and hammering can push peak levels to 95–100 dB or higher, substantially degrading speech intelligibility and team communication. Human studies on speech intelligibility in hospital-like acoustic environments confirm that characteristic hospital noise and concurrent speech markedly reduce correct comprehension of medical sentences, implying that purely verbal, manually enforced checklists are vulnerable to both ambient noise and overlapping speech (Hızıroğlu et al., 2022).

Against this backdrop, automatic speech recognition (ASR) has been explored for “hands-free” checklist verification and intraoperative interaction (Zhou et al., 2022). However, clinical speech differs sharply from the clean, single-speaker audio used to train commercial ASR: accents and code-switching are common, new or rare medical terms appear frequently, homophonous phrases occur in safety-critical slots, and background noise and overlapping talkers are the norm rather than the exception. These factors raise word error rates (WER) and uncertainty for generic ASR systems deployed in OR environments, while leaving the question of “who is speaking” largely unaddressed (Liu et al., 2020). Comparative evaluations already show substantial gaps between general-purpose and domain-specific ASR in both accuracy and fairness across diverse speaker populations, underscoring the need for clinical scenario–specific adaptation and robustness assessment (Madhavaraj et al., 2022).

Early attempts to use voice assistants or smart speakers as timeout aids have shown encouraging results. For example, deep learning–based voice systems for cataract surgery achieved high accuracy in identifying patient information and key procedural elements under both simulated and real conditions, suggesting that speech technology can support surgical safety checks (Feng, 2022; Li et al., 2021). Yet systems that rely only on ASR to convert “what was heard” into “verification passed” remain incomplete. Effective verification requires staged checkpoints (sign-in, time-out, sign-out), clearly assigned responsibilities for surgeons, anesthesiologists, and circulating nurses, and adherence to timing and sequence constraints (Toussaint et al., 2021). Everyday spoken communication adds further uncertainty through synonyms, routine omissions, slips of the tongue, and cross-linguistic expressions. These features call for verification not just at the transcript level, but through structured matching to knowledge rules, consistency checks across phases and roles, and targeted anomaly alerts (Hoebert et al., 2023). Analyses of wrong-site surgery repeatedly highlight “insufficient timeout protocols” and “inadequate team consensus” as central risk factors, rather than mere absence of spoken words.

Building on this body of evidence, we propose and evaluate a knowledge-driven framework for surgical safety checklist verification in noisy, multi-speaker OR environments. The system couples automatic speech recognition with speaker verification (ASR + SV) and a WHO-checklist–based knowledge graph and rule engine. It performs consistency reasoning, conflict resolution, and risk alerts across semantic, role, and procedural dimensions, explicitly modeling who is speaking, what they are saying, and at which checklist phase. The goal is not to replace clinical judgment, but to provide an interpretable, auditable, and robust verification layer that fits within existing team workflows. By reducing missed and erroneous checks without imposing additional manual steps, the framework is intended to strengthen checklist implementation and support a more reliable culture of surgical safety.

## 2 Methods

### 2.1 System overview

**Figure 1:**
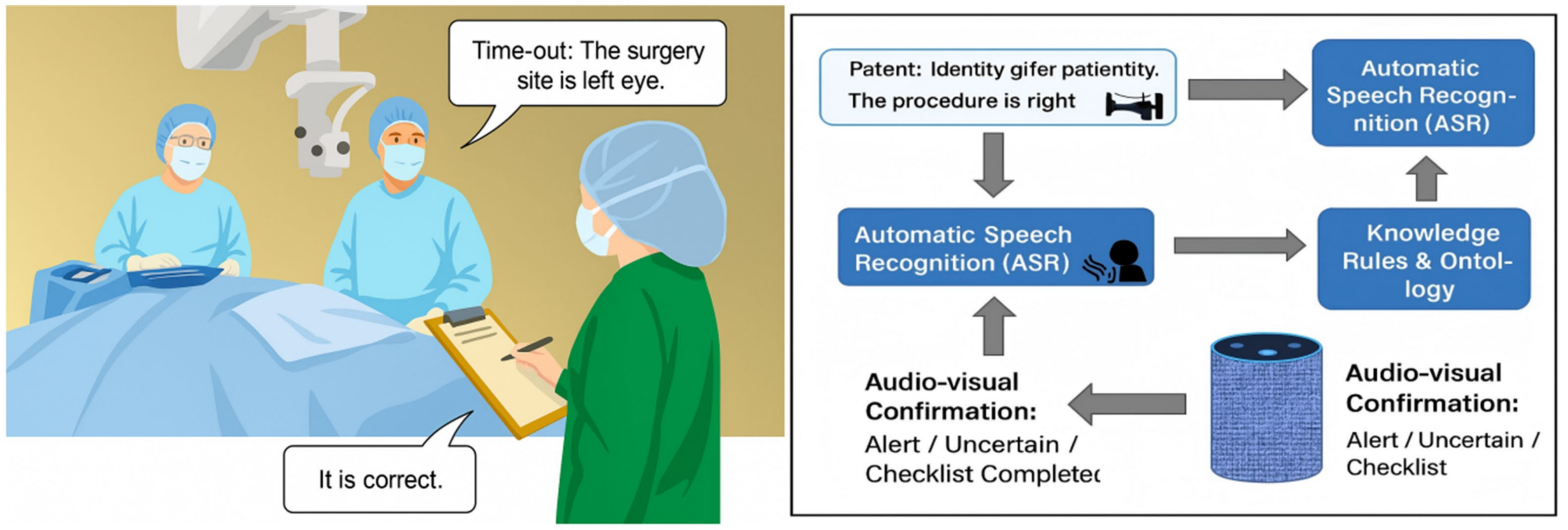
Knowledge-driven framework for integrating speech recognition and speaker verification into surgical safety checks.

Figure 1 illustrates the overall architecture of the knowledge-driven framework for surgical safety verification that jointly uses speech recognition and speaker verification. The central design goal is to provide dual guarantees of semantic alignment and identity reliability for each checklist item.

The left panel of Figure 1 depicts typical verification scenes in which surgeons, anesthesiologists, and circulating nurses verbally confirm the patient’s identity, surgical site, procedure, and critical risk items at the sign-in, time-out, and sign-out phases. The right panel shows the corresponding system pipeline. Multichannel audio from the operating room is captured by a microphone array and passed to the automatic speech recognition (ASR) module, which produces a time-stamped transcript with token-level confidence scores. In parallel, the same audio segments are fed into the speaker verification (SV) module to estimate the likelihood that each utterance was produced by a registered OR team member in a specific role.

The ASR output is then processed by a semantic understanding layer, which identifies checklist entities (e.g., body site, procedure name, patient identifier) and maps them into a structured representation. A knowledge reasoning engine built on the WHO Surgical Safety Checklist ontology validates whether the semantic content, the speaking role, and the checklist phase are mutually consistent in time. After multi-dimensional verification, a knowledge assistant issues audio-visual feedback to the OR team in one of three states: pass (“Checklist complete”), uncertain (recheck required), or alert (potential error). Because the entire process is driven by explicit rules and structured knowledge, every decision is interpretable and traceable, which is essential for deployment in intelligent operating rooms.

**Figure 2:**
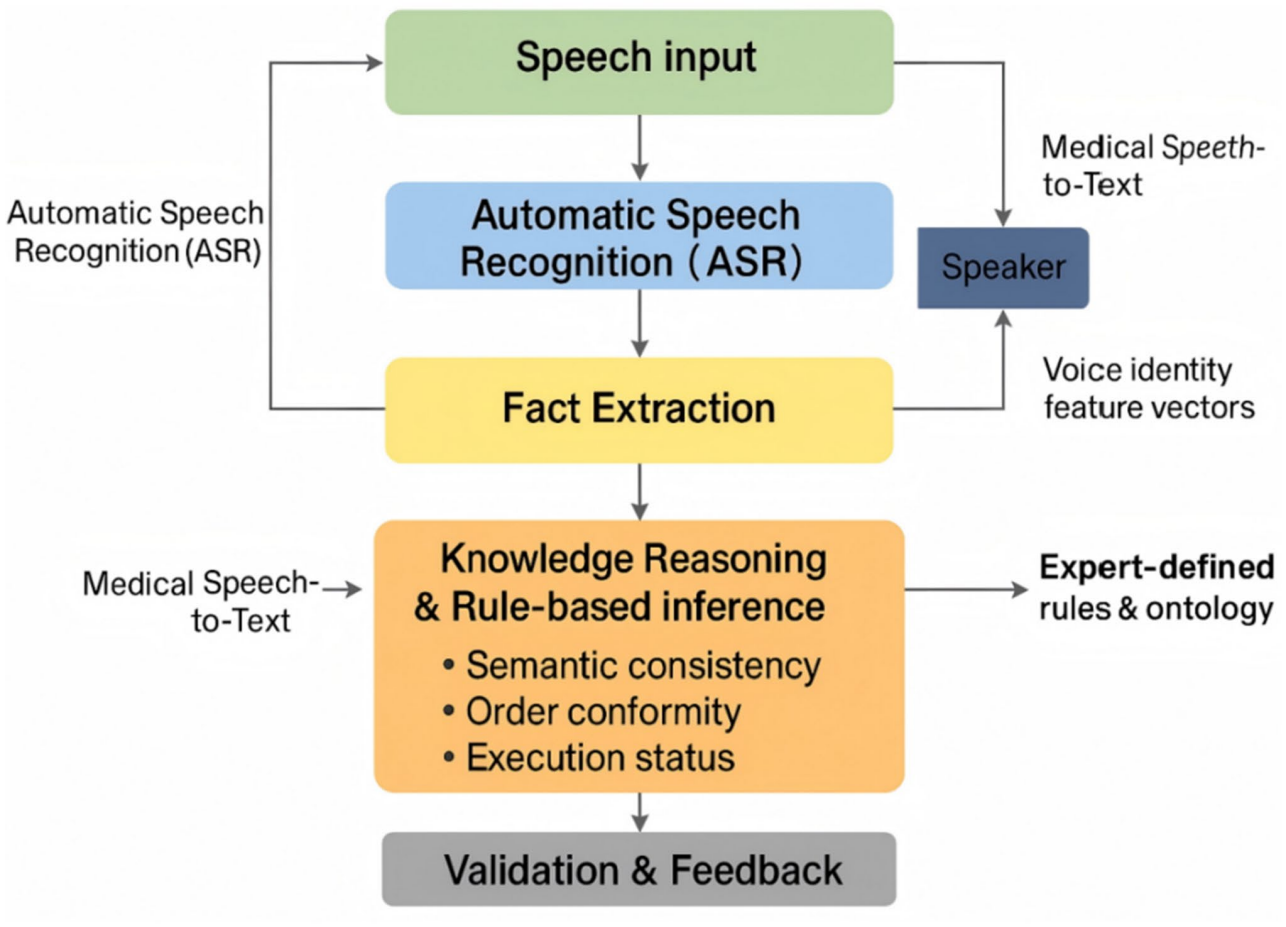
End-to-end workflow of the speech-driven surgical safety check system.

Figure 2 provides a complementary end-to-end view of the signal flow, decomposing the system into five core components: ASR, SV, semantic understanding, knowledge reasoning, and feedback/control. Speech inputs are first transcribed by the ASR module and authenticated by the SV module to ensure that the speaker matches a registered clinical role. A natural language processing (NLP) layer then extracts entities and events from the ASR output using pre-trained embeddings. The knowledge reasoning layer applies rule bases and ontology constraints to check temporal coherence and cross-role consistency. Finally, a verification and feedback layer aggregates decisions over time, logs all events, and drives the visual and spoken prompts to the clinical team.

### 2.2 Dataset

To support training, validation, and evaluation of both ASR and SV components in realistic operating-room conditions, we constructed a dedicated speech corpus for surgical safety checklist verification. The dataset combines simulated surgical dialogs, recorded teaching OR sessions, noise-augmented variants, and controlled overlapping-speech segments to approximate the acoustic and interaction patterns observed in real procedures.

All prompts and dialogs were derived from the WHO Surgical Safety Checklist, with utterances organized around the three canonical phases: sign-in, time-out, and sign-out. Within each phase, scripts covered patient identity confirmation, surgical site and procedure verification, anesthesia risk assessment, antibiotic prophylaxis, equipment and implant checks, and instrument counts. Directional microphones and multichannel audio recorders were used to capture speech while background noise (50–80 dB) from surgical devices and monitors was injected to emulate OR conditions. The corpus includes variations in speaking rate, loudness, and regional accent to improve robustness and generalization.

Each utterance received manual transcription and a two-layer annotation scheme. At the interaction layer, annotators labeled the speaker role (surgeon, anesthesiologist, and nurse), checklist phase, and semantic category (e.g., patient ID, site, procedure, instrument count). At the acoustic layer, they recorded approximate noise level (low/medium/high) and an annotator confidence score for the transcript. Disagreements were resolved by consensus.

For model development, the data were split into training, validation, and test subsets in a 7:1.5:1.5 ratio, with disjoint speakers across splits to avoid identity leakage in both ASR and SV. The composition of the dataset is summarized in Table 1, which lists the main data types, word counts, and storage sizes for each source.

**Table 1:** Composition and labeling scheme of the surgical speech dataset.

Data type	Words (N)	Total Size (MB)	Source	Purpose
Simulated surgical dialog	48,537	623.4	Operating room simulation (multi-speaker)	Model training for ASR + SV
Teaching OR recordings	21,384	312.6	Recorded teaching sessions	Domain adaptation and fine-tuning
Multi-speaker overlap speech	13,156	188.9	Controlled overlapping dialogs	Validation under interference
Whisper and low-volume speech	9,742	156.3	Simulated low-energy utterances	Speech enhancement assessment
Noise-injected augmented data	15,247	262.8	Background noise mixing	Robustness improvement
Cross-accent speech samples	10,958	178.4	Regional accent recordings	Accent adaptation testing
Background noise library	—	96.7	Surgical device and monitor sounds	Data augmentation
Evaluation test set	9,823	142.5	Mixed OR speech samples	Final system benchmarking

### 2.3 Automatic speech recognition (ASR) module

The ASR module is responsible for real-time decoding of spoken checklist content into semantically structured text that can be consumed by the knowledge engine. We implemented a compact end-to-end convolutional neural network (CNN) architecture operating on Mel-spectrogram features and optimized it for deployment on resource-constrained edge devices.

In the preprocessing stage, raw waveforms are resampled, noise-reduced, amplitude-normalized, and segmented using voice activity detection to remove long silences. A short-time Fourier transform (STFT) with a 25 ms window and 10 ms stride is applied, and the resulting spectra are projected onto 40 Mel filter banks to obtain a 2D time–frequency representation. Each input segment is represented as a 40 × 98 × 1 “image” that feeds into the CNN.

The feature extractor consists of stacked convolutional layers with 3 × 3 kernels and batch normalization, interleaved with max-pooling layers that gradually reduce temporal and frequency resolution. The first several convolutional blocks focus on low-level spectral patterns, while deeper blocks capture longer-range coarticulation and prosodic cues. Rectified linear unit (ReLU) activations introduce nonlinearity, and a dropout rate of 0.2 is applied to mitigate overfitting. After the convolutional stack, feature maps are flattened and passed to fully connected layers with a final softmax output over a vocabulary of key phrases and entities relevant to the checklist.

To link ASR output to patient-specific information, recognized tokens are matched against entries from the hospital information system (HIS) and electronic medical record (EMR), such as patient identifiers, scheduled procedures, and planned surgical sites. The ASR module exposes both token sequences and confidence scores to the downstream knowledge reasoning layer, which uses them for semantic validation and decision fusion.

**Figure 3:**
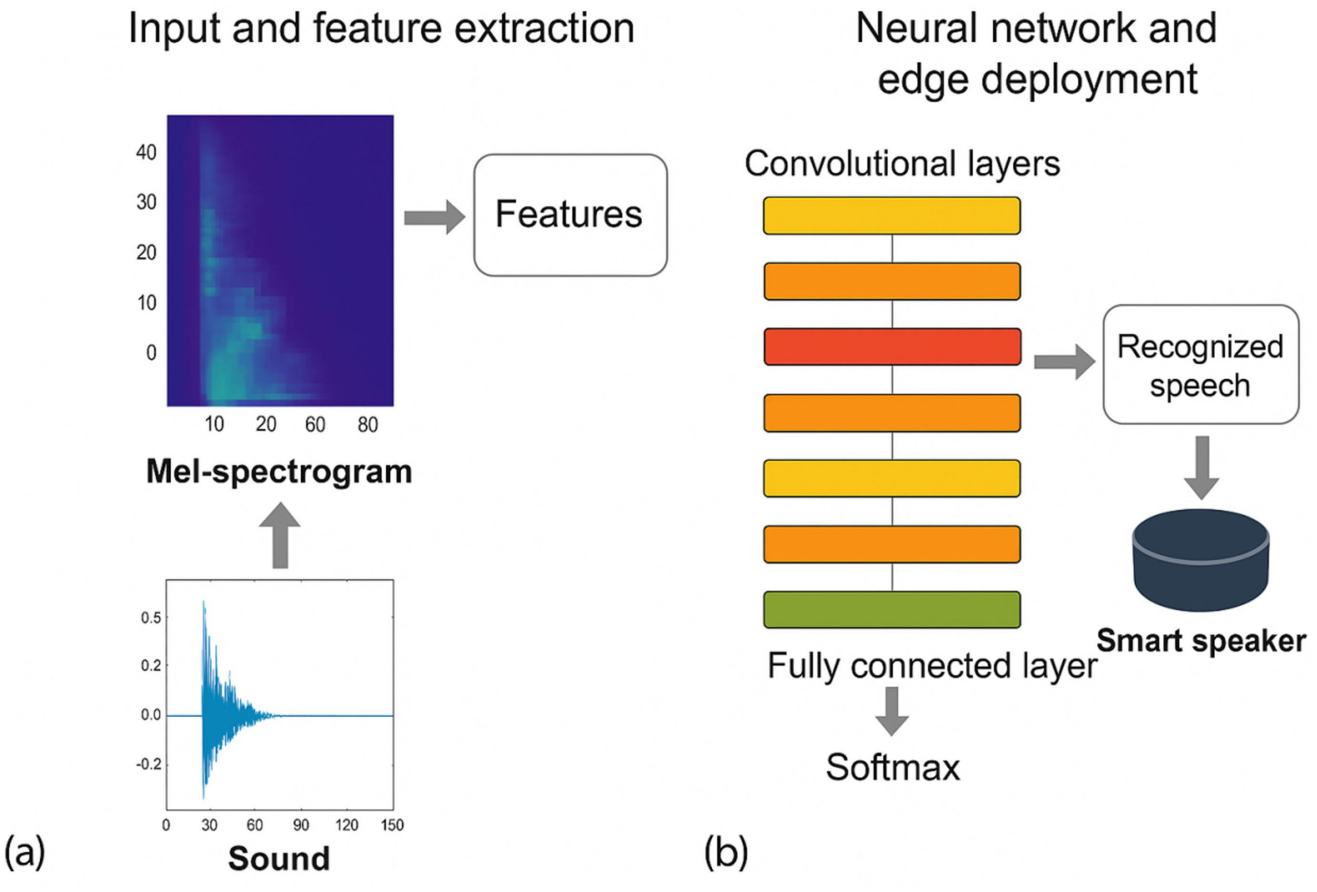
Schematic structure of the ASR module.

The ASR network was implemented in Python using TensorFlow and trained on the corpus described in Section 2.2. The final model contains fewer than 1.3 million parameters and runs in real time on NVIDIA Jetson Nano and Raspberry Pi 4B platforms, with an end-to-end latency below 200 ms for typical utterances. Under medium noise conditions (~70 dB), the system achieved an average word error rate (WER) of 7.4% and a checklist key-phrase accuracy of 91.2%, providing a reliable front end for subsequent knowledge inference and speaker verification (Figure 3).

### 2.4 Speaker verification module

Speaker verification is used to ensure that critical checklist statements are spoken by authorized personnel in the appropriate clinical role. We adopt an ECAPA-TDNN-based SV module, following standard practice for text-independent speaker recognition, and enroll each OR team member (e.g., primary surgeon, anesthesiologist, circulating nurse) prior to the validation sessions.

During enrollment, multiple utterances per speaker are recorded under OR-like conditions and passed through a front-end feature extractor to obtain log-Mel filterbank features. The ECAPA-TDNN network maps each utterance to a fixed-dimensional embedding (speaker vector), and a speaker classifier is trained on the training set to discriminate among enrolled speakers using an additive-margin softmax loss. At inference time, embeddings from enrollment utterances are averaged to form a template for each speaker.

For an incoming utterance, the SV module computes its embedding and measures similarity to each enrolled template using cosine similarity. A verification score is obtained by comparing the similarity to a speaker-specific threshold selected on the validation set to balance false accept and false reject rates. The module outputs both a predicted identity (speaker ID and role) and a confidence score, which are passed to the knowledge reasoning engine.

We evaluate SV performance using the equal error rate (EER), defined as the operating point at which the false acceptance rate equals the false rejection rate, as well as role classification accuracy for checklist utterances. In the integrated system, SV scores are not used in isolation but are combined with ASR confidence and knowledge-rule checks to form the final decision.

### 2.5 Knowledge reasoning and decision fusion

The knowledge reasoning module integrates outputs from ASR, SV, and hospital information systems into a unified decision about each checklist item. It is built on a knowledge base derived from the WHO Surgical Safety Checklist, encoded as an ontology of roles, tasks, and phases and a set of IF–THEN rules that define valid combinations of these elements.

Formally, each event is represented as a triple (role, task, phase) with associated metadata (timestamp, ASR confidence, SV confidence, planned procedure context). Rules specify, for example, which role is authorized to confirm a particular item, in which phase it must occur, and which semantic values are considered correct given the scheduled surgery. Table 2 shows representative rules for surgical-site confirmation and instrument counts.

**Table 2:** Examples of decision-fusion rules for checklist verification.

Rule ID	Input parameters	Decision logic	Example scenario	Output decision
R1	ASR text = “Left eye,” SV = Surgeon	IF semantic = planned site AND speaker = correct role → pass	Surgeon confirms correct eye before incision	Checklist Complete
R2	ASR text = “Right eye,” SV = Surgeon	IF semantic ≠ planned site → alert	Wrong site detected in Time-out	Alert
R3	ASR text = “Left eye,” SV = Nurse	IF semantic = correct but role ≠ authorized → uncertain	Nurse repeats surgical site confirmation	Uncertain
R4	ASR text confidence < 0.75, SV confidence > 0.9	IF text low-confidence AND role verified → recheck	Speech unclear due to background noise	Uncertain (Reconfirm)
R5	ASR text = “All sponges counted,” SV = Nurse	IF phase = Sign-out AND semantic = verified → pass	End of procedure instrument check	Checklist Complete

When both ASR and SV outputs are available for an utterance, the decision fusion engine performs a multi-stage reasoning process:

1. Semantic validation: The ASR transcript is parsed to identify candidate entities (e.g., “left eye”) and mapped to the appropriate ontology slots (e.g., body site). If the semantic value conflicts with the planned procedure (e.g., “right eye” when the EMR lists “left eye”), an alert is raised.
2. Role validation: The SV module’s predicted identity is checked against the authorized role for the current task. If the content is semantically correct but the speaker is not the expected role (e.g., a nurse confirms a site that must be confirmed by the surgeon), the outcome is marked uncertain and a re-prompt is issued.
3. Phase and timing validation: The timestamp and phase label are compared to the expected temporal window for each checklist item. Statements that occur too early or too late can be flagged for confirmation.
4. Confidence fusion: ASR and SV confidence scores are combined using a simple confidence-fusion strategy (e.g., weighted aggregation) to obtain a final decision score. Low ASR confidence but high SV confidence may trigger an uncertain state with a request to repeat, whereas high-confidence semantic and identity matches produce a pass decision.

Examples of such rules are provided in Table 3. For instance, Rule R1 encodes the case where a surgeon correctly confirms the planned left-eye procedure at time-out, leading to a “Checklist complete” decision. Rule R2 captures wrong-site declarations, which generate an alert. Rules R3 and R4 illustrate ambiguous cases in which either the speaking role or the ASR confidence is insufficient, prompting an uncertain decision and recheck.

**Table 3:** Comparative evaluation of models on four surgical safety-check tasks.

Task	AUC	Accuracy (%)	Sensitivity (%)	Specificity (%)
Problem 1: Identity verification (Surgeon vs. Nurse)				
Deep learning (ASR + speaker embedding)	0.993	97.2	96.5	97.8
Random forest	0.982	93.6	92.1	94.3
SVM using RBF kernel	0.967	91.8	90.4	92.5
Problem 2: Procedure confirmation (Cataract vs. Vitrectomy)				
Deep learning (ASR semantic model)	0.991	96.8	95.9	97.2
Random forest	0.978	92.4	91.6	93.5
SVM using RBF kernel	0.962	89.8	88.2	90.7
Problem 3: Time-out stage detection (Time-out vs. Sign-in)				
Deep learning (fusion: ASR + knowledge reasoning)	0.995	98.1	97.3	98.6
Random forest	0.983	94.2	92.8	95.0
SVM using RBF kernel	0.975	90.7	89.5	91.2
Problem 4: Command validation (Checklist complete vs. Uncertain)				
Deep learning (ASR + ontology constraint)	0.989	96.2	95.4	96.9
Random forest	0.977	93.5	92.0	94.3
SVM using RBF kernel	0.959	88.6	86.9	90.1

AUC, area under the receiver operating characteristic curve; ASR, automatic speech recognition; RBF, radial basis function; SVM, support vector machine.

Through this layered reasoning, the module closes the loop from raw speech to semantic understanding and behavioral confirmation. It ensures that automatic verification is both more accurate and more explainable than relying on ASR alone, and that all decisions can be audited post-hoc via structured logs.

### 2.6 Real-time validation protocol

To assess the effectiveness and real-time performance of the proposed knowledge-driven ASR + SV system in a clinically realistic setting, we designed a prospective validation protocol in a simulated operating room. The protocol evaluates performance under multi-speaker interaction, varying noise conditions, and transitions across checklist phases, focusing on speech recognition accuracy, identity verification accuracy, decision consistency, and latency.

Experiments were conducted in a simulation lab configured to approximate the acoustic characteristics of an OR, including a directional microphone array, a single-board computer running the ASR + SV system, and a voice assistant terminal providing feedback. Three surgeons, two anesthesiologists, and three nurses participated after receiving standardized training on the time-out protocol. Each session consisted of scripted sign-in, time-out, and sign-out sequences, repeated under three background-noise conditions: low (< 55 dB), medium (55–70 dB), and high (> 70 dB).

For each utterance, we recorded ASR correctness, SV correctness, the fused system decision (pass/uncertain/alert), and end-to-end response time from speech offset to feedback prompt. Across low- and medium-noise conditions, the system achieved an average response time of 173 ms, with ASR accuracy of 92.4%, SV accuracy of 94.1%, and a decision-level consensus rate of 73.7% with human adjudicators. In high-noise conditions, overall accuracy decreased to 88.6%, but remained higher than the 80.2% achieved by a baseline system without knowledge reasoning. Even under multi-speaker stress and elevated noise, the system maintained real-time responsiveness without noticeable delays or cumulative error propagation over the course of each checklist sequence.

## 3 Results

### 3.1 Quantitative results

**Figure 4:**
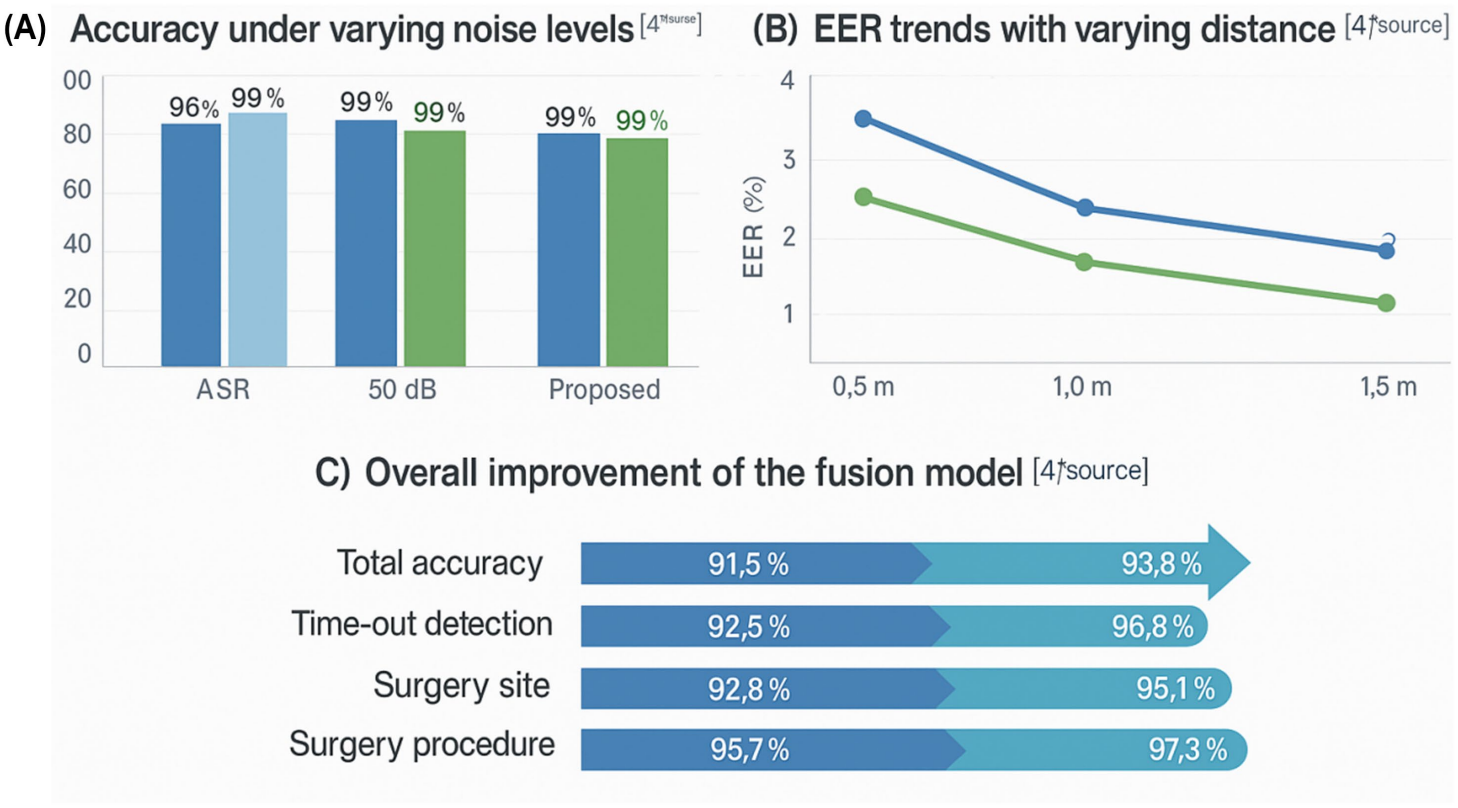
System performance under varying noise levels and distances. **(A)** Checklist accuracy across noise conditions. **(B)** Equal error rate (EER) at different microphone distances. **(C)** Overall improvements of the fusion model relative to the ASR-only baseline.

Figure 4 summarizes the performance of the knowledge-driven fusion model under different noise levels and microphone distances, and compares it with the baseline ASR-only system. Overall, the integrated ASR + SV + knowledge framework maintains high recognition accuracy and a low alarm rate across the tested acoustic conditions, indicating that it is feasible and stable for use in complex operating-room environments.

Figure 4A shows checklist-recognition accuracy across noise levels from 50 to 80 dB. The fusion system achieves point accuracies close to 99%, whereas the ASR-only model plateaus around 96%. This gap widens as background noise increases, suggesting that the additional constraints from speaker identity and knowledge rules help recover correct decisions in acoustically challenging scenarios.

Figure 4B plots the equal error rate (EER) for identity verification at microphone distances between 1.0 and 1.5 m. Across all positions, the fusion model consistently attains lower EER values than the baseline, with typical reductions from approximately 3.7 to 3.2%. These results indicate that the integrated system is less sensitive to changes in speaking distance than a purely acoustic model.

Figure 4C aggregates four task-level metrics to illustrate the overall impact of knowledge-driven fusion. Relative to the ASR-only baseline, overall checklist recognition accuracy increased from 91.5 to 93.8%, time-out detection accuracy improved from 92.5 to 96.8%, surgical-site accuracy rose from 92.8 to 95.1%, and surgical-procedure accuracy increased from 95.7 to 97.3%. Together, these gains show that integrating semantic, identity, and workflow constraints yields consistent performance improvements beyond what can be achieved with ASR alone.

### 3.2 Comparative evaluation across models

To benchmark the proposed framework against more traditional classifiers, we compared three model families on four safety-check tasks: (i) identity verification (surgeon vs. nurse), (ii) procedure confirmation (cataract vs. vitrectomy), (iii) time-out phase detection (time-out vs. sign-in), and (iv) command validation (checklist complete vs. uncertain). Table 3 reports area under the ROC curve (AUC), accuracy, sensitivity, and specificity for a support vector machine with RBF kernel (SVM), a random forest (RF), and deep learning–based models that incorporate ASR features, speaker embeddings, and knowledge reasoning.

Across all four problems, the knowledge-driven deep learning models achieved the best performance. The average AUC of the fusion models was 0.992, compared with 0.980 for RF and 0.966 for SVM. In the time-out detection task, the ASR + knowledge model reached an AUC of 0.995, accuracy of 98.1%, sensitivity of 97.3%, and specificity of 98.6%, indicating strong sensitivity to both semantic content and phase boundaries. For identity verification, the ASR + speaker-embedding model accurately distinguished surgeons from nurses, with an AUC of 0.993 and accuracy above 97%. In the command-validation task, where semantic ambiguity and overlapping speech are common, adding ontology-based constraints reduced false detections while maintaining sensitivity above 95%.

These results show that combining semantic knowledge, speaker identity, and explicit reasoning yields a more discriminative and stable decision process than purely statistical classifiers, particularly in tasks that depend on correct role attribution and phase recognition.

### 3.3 Case demonstration and module-level analysis

**Figure 5:**
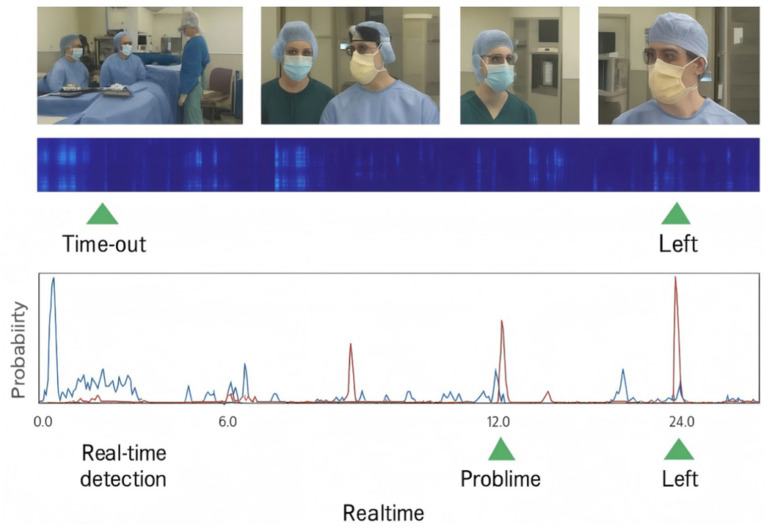
Example of real-time visualization for a surgical safety check sequence.

To illustrate how the integrated system behaves in practice, Figure 5 shows a typical real-time visualization of a safety check sequence. The interface displays the current checklist phase, recognized semantic items (e.g., patient ID, surgical site, procedure), the predicted speaker role, and the system decision state (pass, uncertain, or alert). When an utterance satisfies both semantic and role constraints, the system highlights the item in green and plays a “Checklist complete” confirmation. If semantic content is plausible but the speaker is not the authorized role, the item is marked as uncertain and a recheck prompt is issued. When a discrepancy with the planned procedure is detected, the system raises an alert with both visual and audible warnings.

**Figure 6:**
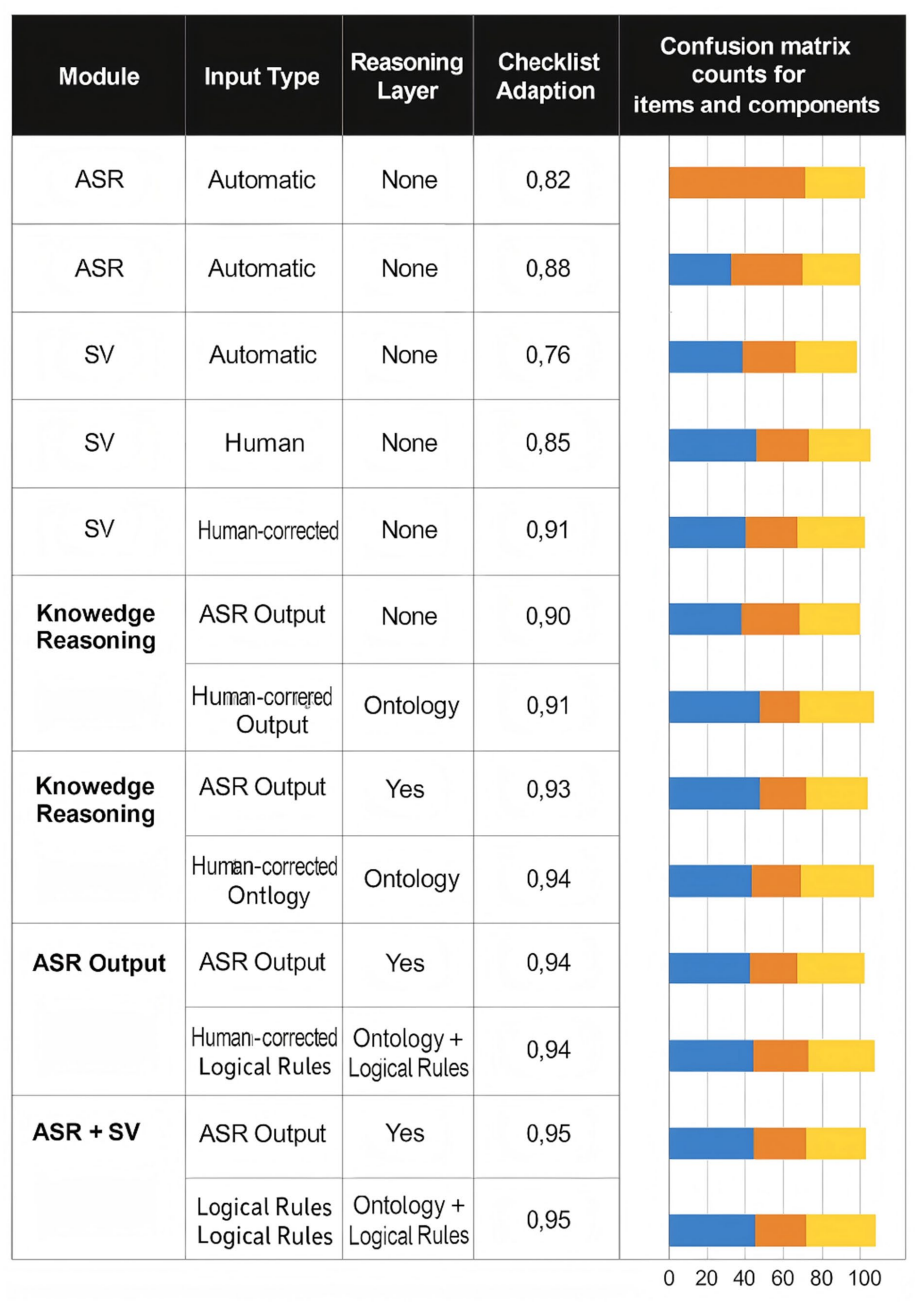
Module-level performance and error patterns for ASR, speaker verification (SV), and knowledge reasoning (KR).

Figure 6 further decomposes performance at the module level by reporting F₁ scores and confusion-matrix patterns for ASR, SV, and the knowledge reasoning (KR) layer under different input conditions. When fed with automated ASR transcripts, the KR layer achieves an F₁ score of 0.95 by integrating both ASR and SV outputs, substantially higher than the standalone ASR (0.82) and SV (0.85) modules. The accompanying error analysis shows that ASR tends to produce more false negatives under high noise, missing some checklist items altogether, while SV is more prone to false positives in dense multi-speaker exchanges. By combining semantic reasoning with explicit rules, the KR layer filters out many of these errors and stabilizes decision-making at the checklist level.

Taken together, the quantitative comparisons and case demonstrations indicate that the proposed fusion model generalizes well across speech conditions and tasks. By jointly leveraging semantic content, speaker identity attributes, and knowledge reasoning, the system delivers accurate, low-error, real-time decisions for surgical verification, supporting the development of interpretable and reliable intelligent surgical safety systems.

## 4 Discussion

The knowledge-driven ASR–SV fusion framework developed in this study offers an intelligent and interpretable approach to preoperative safety verification in noisy, multi-speaker operating rooms (Müller, 2010). Compared with purely text-based or fully manual checks, the system can detect checklist commands in real time, attribute them to specific speakers, and cross-validate them against an explicit knowledge base. By combining speech content, speaker identity, and checklist logic, it reduces the likelihood of missed or erroneous checks and alleviates part of the cognitive load on the surgical team.

At the algorithmic level, the deep learning–based modules outperformed traditional classifiers in both speaker verification and semantic decision tasks (Dong et al., 2026). The compact CNN ASR front end learns time–frequency patterns from Mel-spectrogram inputs and effectively suppresses high-frequency noise through multi-layer convolution and feature pooling (Liu et al., 2024; Liu et al., 2024). Across diverse noise and distance conditions, the integrated framework maintained recognition accuracies above 95%, with relative gains of approximately 3–5 percentage points over the ASR-only baseline (Liu et al., 2025). Reductions in equal error rate (EER) for identity verification further suggest that adding knowledge constraints to fuzzy semantic decisions can correct systematic biases and support logically consistent, explainable outputs.

With respect to robustness, the experimental results indicate that the fusion framework tolerates both elevated noise levels and longer microphone distances. Adaptive acoustic preprocessing, together with rule-based matching, allows dynamic adjustment of decision thresholds and real-time semantic judgments. For example, during time-out confirmation of surgical site and procedure, the system not only considers acoustic confidence but also checks whether the spoken site and procedure are compatible with the planned anatomy recorded in the knowledge base (Medroa Inacio et al., 2025). This joint evaluation reduces the impact of homophonous phrases and overlapping conversations, aligning the system’s decisions more closely with the surgeon’s actual intent and improving semantic robustness in realistic OR conditions (Liu et al., 2025; Gillespie and Ziemba, 2024).

From a clinical applicability perspective, the framework is designed to be embedded into existing operating room information systems without major workflow disruption. Single-board computers and smart speakers can be integrated into the OR network, providing audio-visual prompts during sign-in, time-out, and sign-out. In our validation setting, the system delivered voice responses within 200 ms, which is faster than manual cross-checking by several seconds, and the visual interface made the sequence of verification steps transparent to the team (Turley et al., 2023). This combination of speed and visibility has the potential to support a more reliable safety culture, provided that the technology is introduced with appropriate training and change management.

Several limitations should be acknowledged. First, most of the data used here were obtained from simulated procedures and teaching operating room recordings rather than continuous capture from live surgeries. Although these scenarios mimic key acoustic characteristics, they do not cover the full diversity of dialects, speaking styles, and emergent terminology encountered in daily practice. Second, the current reasoning layer relies primarily on manually designed rules and ontology alignment, without leveraging trainable semantic models that could adapt to more complex contextual variation. Third, the present study did not include formal usability testing with surgeons, anesthesiologists, and nurses, nor did it systematically assess perceptions of trust, workload, or workflow fit. Finally, ethical and legal questions—such as audio-data privacy, long-term storage of voice logs, and responsibility for false alarms or missed alerts—have only been discussed conceptually and require further analysis in collaboration with clinical and institutional stakeholders.

Future work will therefore focus on enlarging and diversifying the corpus with multilingual, multi-accent, and multi-specialty data; integrating learning-based semantic models into the reasoning layer while retaining interpretability; and conducting prospective clinical pilot studies with mixed-method evaluations of user experience and workflow integration. In parallel, we plan to develop guidelines for secure data handling and to explore how audit logs from the system can be used in morbidity and mortality reviews and quality-improvement programs.

## 5 Conclusion

This study presents a knowledge-driven, voice-based verification framework for surgical safety checks that combines deep learning–powered ASR, speaker verification, and explicit medical knowledge reasoning. The system identifies and validates critical checklist items—such as verification commands, surgical sites, and procedures—under multi-noise, multi-distance, and multi-speaker conditions. Compared with baseline methods, the integrated model achieved higher accuracy and specificity, including 98.1% accuracy and 98.6% specificity during time-out verification, while maintaining real-time responsiveness suitable for embedded deployment.

By delivering synchronized voice responses and visual prompts, the framework provides traceable and reproducible evidence for each verification step and helps reduce the risk of human error. At the same time, its current validation is based largely on high-fidelity simulations and limited real-world testing, without formal user and ethical evaluation. The findings thus support the feasibility of AI-driven speech detection for surgical environment verification, while also highlighting the need for larger clinical studies, broader corpus development, and more comprehensive consideration of privacy, accountability, and workflow acceptance in future deployments of intelligent, knowledge-driven safety systems.

## Data Availability

The original contributions presented in the study are included in the article/supplementary material, further inquiries can be directed to the corresponding author/s.
